# *Staphylococcus aureus* Decreases SUMOylation Host Response to Promote Intramacrophage Survival

**DOI:** 10.3390/ijms22158108

**Published:** 2021-07-28

**Authors:** Nadhuma Youssouf, Clara Recasens-Zorzo, Virginie Molle, Guillaume Bossis, Philippe Soubeyran, Laila Gannoun-Zaki

**Affiliations:** 1 Laboratory of Pathogen Host Interactions, Université de Montpellier, CNRS, UMR 5235, 34000 Montpellier, France; nadhuma.youssouf@umontpellier.fr (N.Y.); virginie.molle@umontpellier.fr (V.M.); 2 IGMM, Univ Montpellier, CNRS, 34293 Montpellier, France; clara.recasens@igmm.cnrs.fr (C.R.-Z.); guillaume.bossis@igmm.cnrs.fr (G.B.); 3 Centre de Recherche en Cancérologie de Marseille (CRCM), INSERM U1068, CNRS UMR 7258, Aix-Marseille, Université and Institut Paoli-Calmettes, Parc Scientifique et Technologique de Luminy, 13009 Marseille, France; philippe.soubeyran@inserm.fr

**Keywords:** *Staphylococcus aureus*, SUMOylation, infection, Ubc9, intracellular survival, macrophage

## Abstract

*Staphylococcus aureus* is a commensal bacterium that causes severe infections in soft tissue and the bloodstream. During infection, *S. aureus* manipulates host cell response to facilitate its own replication and dissemination. Here, we show that *S. aureus* significantly decreases the level of SUMOylation, an essential post-translational modification, in infected macrophages 24 h post-phagocytosis. The reduced level of SUMOylation correlates with a decrease in the SUMO-conjugating enzyme Ubc9. The over-expression of SUMO proteins in macrophages impaired bacterial intracellular proliferation and the inhibition of SUMOylation with ML-792 increased it. Together, these findings demonstrated for the first time the role of host SUMOylation response toward *S. aureus* infection.

## 1. Introduction

*Staphylococcus aureus* is a highly adaptable human pathogen, leading to various nosocomial and community-acquired infectious diseases [1,2]. *S. aureus* can invade a variety of non-professional phagocytes and can survive within professional phagocytes during several days [3,4,5]. During infection, pathogen bacteria exploit several eukaryotic signaling pathways and manipulate their host cells to allow their own replication, propagation, and escape from host immune responses [6]. Post-translational modifications (PTMs) play a key role in the functional proteomic as they regulate the activity, localization, and interaction with cellular molecules such as nucleic acids, lipids, cofactors, and other proteins. PTMs include phosphorylation, acetylation, methylation, and also the addition of small polypeptides such as ubiquitin or ubiquitin-like proteins such as the Small Ubiquitin-like Modifier (SUMO). Whereas several pathogens are known to use PTMs for their own benefit [7,8,9,10,11], only few pathogenic bacteria have been reported to interfere with the SUMOylation processes [12,13,14,15,16]. SUMOylation is a reversible eukaryotic PTM in which SUMO is covalently linked to the lysine residues of target proteins. It is an essential mechanism regulating cellular processes such as DNA replication, transcription, RNA processing, and cell signaling [17,18]. In mammals, there are three SUMO proteins, SUMO1, SUMO2, and SUMO3, with SUMO2 and SUMO3 being almost identical. The SUMOylation machinery consists of a set of different enzymes: the SUMO-activating enzyme E1, which is a heterodimer of the SAE1/SAE2 subunits, the E2 conjugating enzyme Ubc9, and several E3 ligases. The SUMO regulatory pathway has been associated with many different diseases such as cancer, neurological and metabolic disorders, as well as response to infection [19,20]. Although *S. aureus* is able to induce DNA damage of its host cells [21], and modify the host global protein activity [22,23], the role of SUMOylation regarding intracellular *S. aureus* survival was still unknown. In this study, we show for this first time that *S. aureus* infection leads to a massive decrease in SUMOylation in infected macrophages, participating in *S. aureus* intracellular survival and proliferation.

## 2. Results

### 2.1. S. aureus Infection Decreases Macrophages SUMOylated Proteins

**Figure 1:**
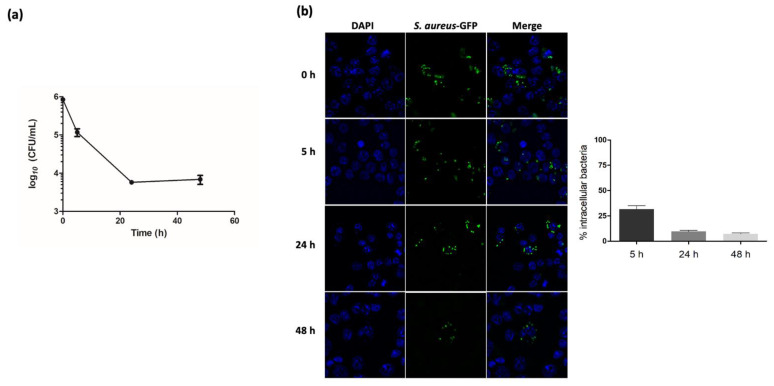
*S. aureus* strain NSA739 persists intracellularly in Raw264.7 macrophages up to 48 h post-gentamicin treatment (pGt). (**a**) Macrophages were infected with *S. aureus* NSA739 strain expressing the GFP protein at an MOI of 10 for 1 h, and the number of gentamicin-protected bacteria was determined at different time points by plating intracellular bacteria for CFU enumeration. (**b**) Representative images of fluorescence microscopy of GFP-labeled *S. aureus* NSA739. Macrophages seeded on coverslips were infected with GFP-labeled bacteria at a MOI of 10 and analyzed at 5, 24, and 48 h pGt using a 63-oil objective. Quantification was performed using intracellular bacteria at T0 pGt as 100% and is the result of independent counting of 100 cells from each of three independent experiments. n.i, non-infected control cells.

The success of *S. aureus* as a pathogen is partly due to its ability to survive and proliferate within different cell types, including macrophages. As expected, we confirmed by counting intracellular bacteria using CFU numeration and fluorescence microscopy that the *S. aureus* NSA739 clinical strain can survive in Raw264.7 macrophages up to 48 h post phagocytosis (Figure 1a,b).

**Figure 2:**
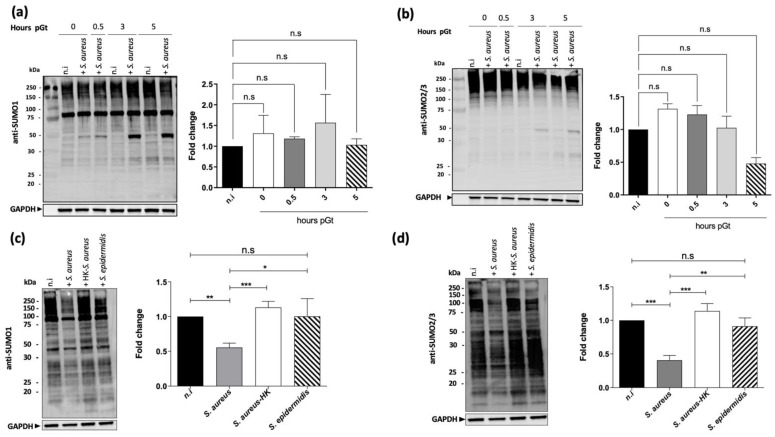
*S. aureus* decreases SUMOylation after long-term macrophage infection. Immunoblot analysis of SUMO1 (**a**), SUMO2/3 (**b**), and GAPDH levels in lysates of macrophages infected with *S. aureus* for different time points up to 5 h pGt. SUMO1 and SUMO2/3 smears were quantified from four independent experiments using Image lab software (ChemiDoc) and normalized to GAPDH (right panels). The fold change graph represents the percentage of SUMOylated proteins by SUMO1 obtained in the infected cells compared to the amount of SUMOylated proteins in the non-infected control macrophages. The data presented are the mean ± SD of three independent biological experiments. n.s, not significant (Kruskal–Wallis test followed by Dunn’s post hoc test). (**c**,**d**) Immunoblot analysis of SUMO1 (**c**), SUMO2/3 (**d**) and GAPDH levels in lysates of macrophages infected with *S. aureus*, heat-killed *S. aureus* and *S. epidermidis* for 24 h pGt. n.i., non-infected control cells. SUMO1 and SUMO2/3 smears were quantified from four independent experiments using Image lab software (ChemiDoc) and normalized to GAPDH (bottom panels). The fold change graph represents the percentage of SUMOylated proteins by SUMO1 obtained in the infected cells compared to the quantity of SUMOylated proteins in the non-infected control cells (right panels). *** *p* ≤ 0.001; ** *p* ≤ 0.01; * *p* ≤ 0.05 by one-way ANOVA with Bonferroni’s multiple-comparison test.

We next thought to investigate if *S. aureus* alters the SUMOylation of cellular proteins in macrophages as a strategy to survive intracellularly. Then, we compared the global pattern of SUMO1- and SUMO2-/3-conjugated protein levels in Raw264.7 cells uninfected or infected with *S. aureus* up to 5 h pGt (post-gentamicin treatment). Surprisingly, and unlike intracellular bacteria such as *Listeria* and *Salmonella* [14,16], we observed that up to 5 h pGt, the global pattern of SUMO-conjugated proteins was similar in infected and non-infected cells (Figure 2a,b). Then, we decided to investigate whether *S. aureus* modify the host cell SUMOylation profile after long-term infection. Since an identical number of viable intracellular bacteria was recovered at 24 h and 48 h pGt, infections with either *S. aureus*, heat-killed *S. aureus*, or the coagulase negative strain *S. epidermidis* were performed at 24 h pGt. Quantification after 24 h pGt revealed a significant and specific decrease of SUMO1 (Figure 2c) and SUMO2/3 (Figure 2d) modified proteins in macrophages infected with *S. aureus* NSA739 strain compared to cells infected with heat-killed *S. aureus* or *S. epidermidis* bacteria that remained similar to the non-infected SUMO-profile. Moreover, similar results were obtained when Raw264.7 cells were infected with the *S. aureus* Newman strain (data not shown), indicating that the decrease of the SUMOylated proteins was not restricted to the clinical NSA739 strain. On the other hand, we also explored the SUMOylome in non-phagocytic cells. For this purpose, HEK293 cells (Human Embryonic Kidney cells) were infected with the NSA739 strain over time and SUMO2-conjugated proteins levels were compared with the non-infected cells. Similar to the results obtained in the Raw264.7 cells, the SUMO2-conjugated proteins decreased in the infected HEK298 cells. However, these cells were more sensitive to the infection with *S. aureus* after 24 h pGt than the Raw264.7 cells, preventing us from following long-term infections in these cells. All together, these findings suggest that this SUMOylome decrease is specific to live and coagulase-positive *S. aureus* strains in phagocytic and non-phagocytic infected cells.

### 2.2. Host SUMOylation Diminishes S. aureus Intramacrophagic Replication

**Figure 3:**
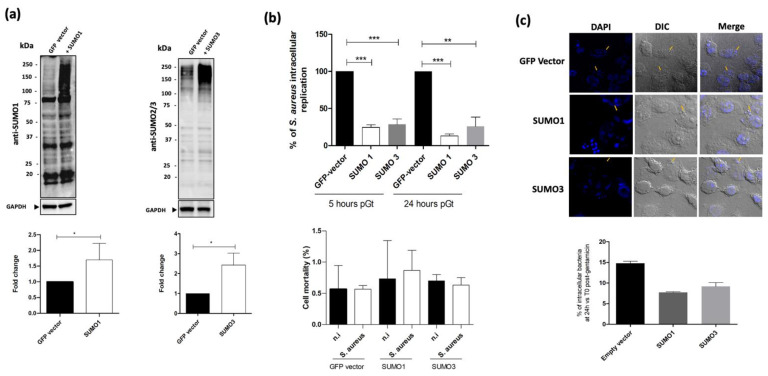
SUMOylation over-expression decreases the intracellular survival of *S. aureus*. (**a**) Immunoblot analysis of SUMO1 (left panel), or SUMO2/3 (right panel) over-expressing macrophages versus control macrophages (GFP vector). GAPDH levels in lysates were used to standardize protein amounts. SUMO1 and SUMO2/3 smears were detected using Image lab software (ChemiDoc) and normalized to GAPDH (bottom panels). (**b**) Intracellular *S. aureus* number recovered from macrophages overexpressing SUMO was counted after cell lysis and is presented as the ratio of intracellular bacteria at 5 h and 24 h post-gentamicin compared to cells transfected with an empty GFP vector, considered as 100%. Cell mortality was assessed using trypan blue exclusion dye 0.4% (*w*/*v*) to evaluate the number of blue (dead) cells. (**c**) Representative confocal images of infected macrophages with *S. aureus* for 24 h pGt. Arrows show intracellular bacteria. The percentage of intracellular bacteria in macrophages overexpressing SUMO1, SUMO3, or GFP vector is represented as the ratio of intracellular bacteria at 24 h vs. T0 pGt and is the result of independent counting of 100 cells from each of three independent experiments. DIC: differential interference contrast. *** *p* ≤ 0.001; ** *p* ≤ 0.01; * *p* ≤ 0.05 by one-way ANOVA with Bonferroni’s multiple-comparison test.

To further characterize the role of the host SUMOylation response toward *S. aureus* infection, we artificially increased the level of SUMOylated proteins in Raw264.7 macrophages by over-expressing SUMO1 or SUMO3 using lentivirus vectors [24] as confirmed by immunoblots (Figure 3a). Then, macrophages over-expressing SUMO1 or SUMO3 were infected with *S. aureus*, and the number of viable intracellular bacteria was determined 24 h pGt. The cellular viability of these cells was estimated to be over 95% using two different methods, the trypan blue exclusion dye and the lactate dehydrogenase (LDH) release (data not shown). Our results show that the over-expression of either SUMO1 or SUMO2 significantly reduced *S. aureus* intracellular replication at both 5 h and 24 h pGt compared to control macrophages expressing GFP (Figure 3b). Moreover, confocal microscopic analysis 24 h pGt confirmed the decrease of intracellular *S. aureus* bacteria in SUMO-overexpressing macrophages (Figure 3c). Taken together, these data reveal that host cells SUMOylation increase is detrimental to *S. aureus* intramacrophagic survival.

**Figure 4:**
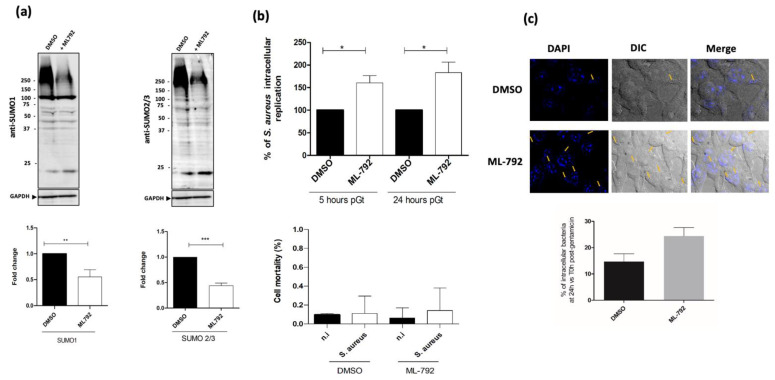
SUMOylation inhibition increases the intracellular survival of *S. aureus*. (**a**) Immunoblot analysis of Raw264.7 macrophages were pretreated with ML-792 at 0.5 µM or DMSO as a control. SUMO1 and SUMO2/3 smears were quantified and normalized to GAPDH (bottom panels). (**b**) Macrophages pretreated with ML-792 at 0.5 µM or DMSO were infected with *S. aureus*. The number of intracellular bacteria recovered from macrophages after 5 h or 24 h post-gentamicin was counted and is presented as the ratio of intracellular bacteria compared to cells pretreated with DMSO, which is considered as 100%. Cell mortality was assessed using trypan blue exclusion dye 0.4% (*w*/*v*) to evaluate numbers of blue (dead) cells. (**c**) Representative confocal images from cells infected with *S. aureus* NSA739 for 24 h pGt using a 63-oil objective. Arrows show intracellular bacteria. The percentage of intracellular bacteria in pretreated cells with ML-792 is represented as the ratio of intracellular bacteria from ML-792 pretreated cells over DMSO-pretreated cells at 24 h pGt. The quantification is the result of independent counting of 100 cells from each of three independent experiments. DIC: differential interference contrast. *** *p* ≤ 0.001; ** *p* ≤ 0.01; * *p* ≤ 0.05 by one-way ANOVA with Bonferroni’s multiple-comparison test.

In addition, the role of SUMOylation in the control of *S. aureus* intracellular replication was investigated in macrophages pretreated with ML-792, an inhibitor of the SAE1/SAE2 enzyme [25]. As expected, macrophages treated with the ML-792 inhibitor showed a strong diminution of SUMOylated proteins (Figure 4a). Interestingly, in such SUMOylation-deficient macrophages, we observed a significant increase of *S. aureus* intracellular replication at 5 h and 24 h pGt (Figure 4b,c). Our results show that the SUMOylation-dependent macrophage response represents an important host defense mechanism in order to reduce *S. aureus* intracellular survival at late post-phagocytosis times.

### 2.3. The Level of the SUMOylation Enzyme Ubc9 Is Reduced upon S. aureus Infection

**Figure 5:**
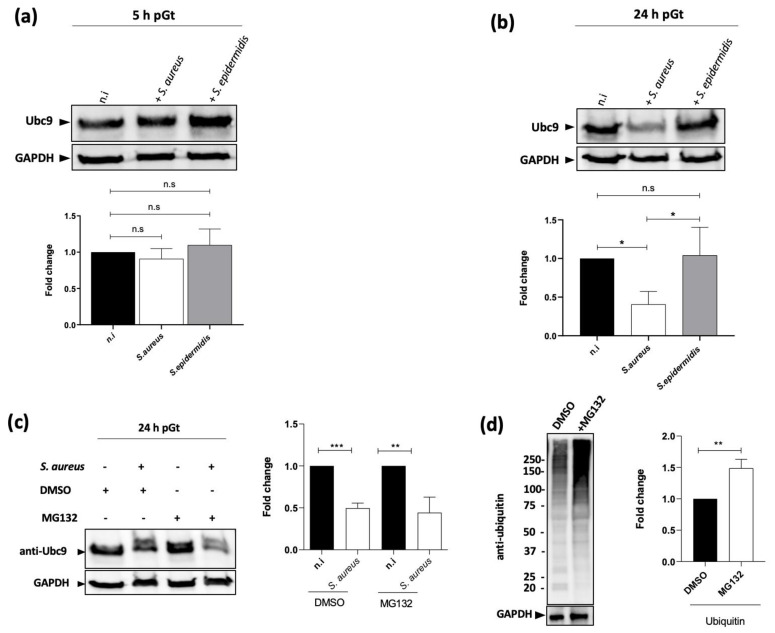
*S. aureus* decreases the Ubc9 protein level in a proteasome-independent pathway. Immunoblot analysis of Ubc9 and GAPDH levels in lysates of macrophages infected with *S. aureus* and *S. epidermidis* for 5 h (**a**) and 24 h (**b**) post-gentamicin treatment. n.i., non-infected control cells. h, hour post-gentamicin treatment. Ubc9 bands were quantified from three independent experiments and normalized to GAPDH levels. The graph represents fold change compared to non-infected cells at 5 h and 24 h post-gentamicin. n.s.: non-significant; * *p* ≤ 0.05 by one-way ANOVA with Bonferroni’s multiple-comparison test. (**c**,**d**) Immunoblot analysis of Ubc9 (**c**), ubiquitin-conjugated proteins (**d**) in macrophages pretreated or not with MG132 for 3 h prior to infection with *S. aureus*. Bands were quantified from three independent experiments using Image lab software (ChemiDoc) and normalized to GAPDH. The fold change graph represents the percentage of Ubc9 and ubiqutiin-conjugated proteins obtained in the infected cells compared to the quantity of proteins in the cells treated with DMSO (right panels). *** *p* ≤ 0.001; ** *p* ≤ 0.01; * *p* ≤ 0.05 by one-way ANOVA with Bonferroni’s multiple-comparison test.

Having established that the inhibition of SUMOylation in macrophages is an essential mechanism for *S. aureus* intracellular survival, we decided to investigate how *S. aureus* was able to achieve this regulation. The SUMOylation reaction is dependent on several enzymes such as SAE1/SAE2, Ubc9, and E3 ligases, allowing SUMOs activation and transfer to the lysine residues of specific substrates [18]. Therefore, in order to identify how *S. aureus* could impair the host SUMOylation machinery, we quantified the level of the Ubc9 enzyme in macrophages infected, either with *S. aureus* or the coagulated negative strain *S. epidermidis*. As shown in Figure 5a, no significant difference in Ubc9 protein level was observed at 5 h pGt between *S. aureus* and *S. epidermidis* infected macrophages. However, in accordance with the SUMOlation profiles (Figure 2c,d), Ubc9 protein level in infected macrophages with *S. aureus* showed a decrease around 50% at 24 h pGt as compared to uninfected cells, while no significant decrease was observed in *S. epidermidis* infected cells. (Figure 5a,b). Our results suggest that the level of the Ubc9 enzyme is involved in the survival of *S. aureus* strains by lowering SUMOylation response of infected macrophages at late time of infection compared to the *S. epidermidis* gender that is in general less virulent than *S. aureus* as a result of lower capacity to survive after macrophages phagocytosis.

In order to investigate the role of the proteasome in the decrease of Ubc9 level, we used the proteasome inhibitor MG132. Inhibition of proteasome activity by MG132 has no effect on the level of Ubc9 (Figure 5c), which still decreased in infected macrophages 24 h pGt. As a control, we checked that the amount of ubiquitin-conjugated proteins should significantly increase as expected in macrophages treated with MG-132 (Figure 5d), and cellular integrity was checked by LDH quantification. These data suggest that the decrease of Ubc9 level after infection is proteasome-independent.

## 3. Discussion

Post-translational modifications are involved in transcriptional regulation, stress responses, and DNA damage, among several other cellular functions. Pathogen microorganisms possess the remarkable ability to exploit post-translational modification pathways to promote their own survival and propagation. While viruses have been reported to modulate SUMOylation [26,27], there are only a few reports describing the role of SUMOylation in bacterial intracellular survival. The impact of bacterial infection on this post-translational modification remains poorly studied [12,15]. The mechanisms by which pathogenic bacteria modulate host protein SUMOylation have only been recently investigated and remain poorly understood. Some pathogens are able to target enzymes from the host SUMOylation machinery to modify their activity. The enteropathogenic bacteria Salmonella typhimurium reduces the SUMOylation host response through the upregulation of two microRNA that post-transcriptionally repress Ubc9 [16]. *Shigella flexneri* modifies SUMO-conjugated proteins involved in epithelial invasion and the control of mucosal inflammation [13,28]. More recently, the adherent-invasive *E. coli* (AIEC) bacteria were shown to be able to inhibit autophagy by modifying host SUMOylation allowing intracellular replication [12]. *Klebsiella pneumoniae* is able to decrease SUMOylation to promote infection by limiting the activation of host inflammatory responses [15]. *S. aureus* is able to survive intracellularly by hijacking host cell defenses [6], but the putative role of host SUMOylation after *S. aureus* infection was unknown. In this study, we demonstrated that *S. aureus* induces a decrease of macrophages SUMOylation response, thus promoting its intracellular survival. Moreover, the clinical *S. aureus* NSA739 strain, but not the coagulase negative *S. epidermidis* strain, reduces specifically and significantly SUMO1 and SUMO2/3-conjugates 24 h post-infection.

Our results are in accordance with previous studies reporting that some intracellular pathogens survival involved the modification of the host SUMOylation response [12,13,14,16]. Interestingly, *S. aureus* impact on SUMOylation host response occurs at longer times post-infection and then seems critical for bacterial long-term persistence.

Moreover, we showed (i) that the over-expression of SUMO1 or SUMO3 in macrophages host cells reduces *S. aureus* survival, thus confirming the role of this SUMO-dependent regulation, (ii) that when macrophages were treated with the SUMOylation inhibitor, ML-792, known to inhibit the E1 enzyme SAE1/SAE2 [25], *S. aureus* was then able to proliferate at a higher rate in such SUMO-inhibited macrophages, and (iii) that when macrophages were infected with *S. aureus*, the level of Ubc9 was significantly reduced in infected macrophages 24 h pGt. These results suggest that *S. aureus* induces a general deSUMOylation in host cells at the late infection stage, at least through the decrease of the Ubc9 protein, to favor its intracellular replication. Moreover, MG132 proteasome inhibitor had no effect on Ubc9 protein level. Thus, the mechanism underlying Ubc9 degradation such as cellular or lysosomal proteases remains to be determined.

However, the precise mechanism of action remains to be elucidated. It has been reported that pathogens such as *Xanthomonas euvesicatoria* [29] or *Yersinia pestis* [30] secrete effectors able to mimic host deSUMOylases, therefore inducing a global deSUMOylation. *Listeria monocytogenes* alters host SUMOylation by degrading Ubc9 through the action of the pore-forming toxin listeriolysin (LLO) [14]. Regarding *S. aureus*, we could hypothesize that upon infection, secreted virulence effectors could interfere with the host SUMOylation response to allow long-term intracellular survival. However, such *S. aureus* effectors remain to be identified.

In conclusion, in the present study, we demonstrated for the first time that *S. aureus* impairs protein SUMOylation via the decrease of Ubc9 conjugation enzyme in order to promote its survival and persistence upon long-term infection.

## 4. Materials and Methods

### 4.1. Bacterial Strains and Growth Conditions

The clinical *S. aureus* strain NSA739 [31] and the *Staphylococcus epidermidis* strain were plated on Tryptic Soy Agar (TSA) or grown in Tryptic Soy Broth (TSB) medium at 37 °C under agitation. *S. aureus* NSA739 bacteria were heat-killed at 80 °C for 20 min.

### 4.2. Macrophages Culture and Infection

The murine macrophage cell line Raw264.7 (mouse leukemic monocyte macrophage, ATCC TIB-71) was maintained in Dulbecco’s modified Eagle’s medium (ThermoFisher Scientific, Life Technologies Europe, Kwartsweg 2, Bleiswijk, The Netherland) supplemented with 10% of fetal bovine serum at 37 °C in a humidified atmosphere at 5% CO2. Raw264.7 cells expressing 6His-tagged SUMO1 and SUMO3 proteins were generated using lentiviral transduction, as previously described [24]. For macrophage infection, cells were seeded at 5 × 10^5^ cells/mL 24 h before infection and infected at a multiplicity of infection (MOI) of 10:1 (bacteria:cells). Lysis of macrophages and enumeration of intracellular bacteria at time points T0, T0.5 h, T5 h, T24 h, or T48 h post-gentamicin treatment (pGt) was performed as described previously [22].

### 4.3. Fluorescent Microscopy

Cells seeded on coverslips (ThermoFisher Scientific, 1000 stück Deckgläser, Varrentrappstraße 5, Braunschweig, Germany) were infected with the *S. aureus*-GFP strain at an MOI of 10 for 1 h, washed with PBS, and the culture medium containing 100 µg/mL was added for 30 min. Then, cells were incubated in lysostaphin-containing media for different time points. Thereafter, cells were fixed with 4% paraformaldehyde followed by washing in PBS. Then, coverslips were mounted with one drop of Vectashield DAPI (diamidino-2-phenylindole, Vector Laboratories, Inc., Burlingame, CA 94010, USA) and slides were analyzed with a fluorescent microscope (Leica Thunder, Montpellier Ressources Imagerie platform, Montpellier, France) using a 63-oil objective.

### 4.4. Immunoblotting

Infected macrophages were lysed in 100 µL of 2.5X Laemmli buffer boiled for 10 min at 95 °C, sonicated for 10 s at 50% amplitude (DIGITAL Sonifier, Model 450-D, BRANSON), and centrifuged for 1 min at 12,000× *g*. Proteins were resolved on SDS-PAGEs, transferred to PVDF membranes, and subjected to Western blot analyses using an anti-SUMO1 or anti-SUMO2/3 antibody as primary antibody and an HRP-coupled donkey-anti-mouse antibody as secondary antibody (Jackson ImmunoResearch, Interchim, Montluçon, France). SUMO-1 (21C7) and SUMO-2 (8A2) hybridomas were from the Developmental Studies Hybridoma Bank. The immunoblots were detected with the Enhanced Chemiluminescence Detection kit (ChemiDoc^TM^, BioRad Laboratories, Inc, Irvine, CA, USA) and quantified using Image Lab software (BioRad Laboratories, Inc, Irvine, CA, USA).

### 4.5. Cell Viability

Two methods were performed to evaluate cell viability; (i) trypan blue exclusion dye 0.4% (*w*/*v*) (#T6146, Sigma-Aldrich, Life Technologies Corporation, Grand Island, NE, USA) in PBS was added in a ratio of 1:1 to the wells, incubated for 10 min, and gently washed with PBS. Subsequently, the cells were observed under light microscopy to evaluate numbers of blue (dead) cells. The graphs present the percentage of death cells in infected and non-infected cells; (ii) the release of lactate deHydrogenase (LDH) was measured using the Invitrogen CyQuant LDH Cytotoxicity Assay Kit (ThermoFisher Scientific, RockFord, IL, USA) according to the manufacturer’s instructions. Measure of absorbance was performed with the Tecan apparatus (Tecan, Model Spark, Grödig, Austria GmbH).

### 4.6. Statistical Analyses

The statistical significance of changes between groups was determined using the GraphPad software package Prism 6.01. *p* values < 0.05 were considered statistically significant.

## Data Availability

The datasets generated during and/or analyzed during the current study are available from the corresponding author on reasonable request.
